# Which animal-to-feeding-place ratio at time-controlled hay racks is animal appropriate? Preliminary analysis of stress responses of horses

**DOI:** 10.3389/fvets.2022.1005102

**Published:** 2023-01-09

**Authors:** Miriam Baumgartner, Michael H. Erhard, Margit H. Zeitler-Feicht

**Affiliations:** ^1^Agroscope, Swiss National Stud Farm, SNSTF, Avenches, Switzerland; ^2^Department of Veterinary Sciences, Faculty of Veterinary Medicine, Animal Hygiene and Animal Husbandry, Chair of Animal Welfare, Ethology, Ludwig-Maximilians-University Munich, Munich, Germany; ^3^Ethology, Animal Husbandries and Animal Welfare Research Group, Chair of Organic Agriculture and Agronomy, TUM School of Life Sciences, Technical University of Munich, Freising, Germany

**Keywords:** equine welfare, horse husbandry, group housing, feeding system, salivary cortisol, aggressive behavior, quantitative behavior assessment, affective state

## Abstract

**Introduction:**

Automated feeding systems offer numerous advantages for animals and humans, but the associated benefits and risks can often only be seen under practical conditions. The space allowance (~80 cm per horse) at time-controlled hay racks for horses in group housing, unlike at partitioned feeding stands or transponder-controlled feed stations, currently falls below the required individual distance between the horses, which can result in a high level of aggression between the horses due to the lack of partitions between them. Hence, a feed-through at a time-controlled hay rack cannot be equated with a feeding place. In this preliminary study, we therefore aimed to determine the minimum animal-to-feeding-place ratio (AFR) at time-controlled hay racks that would provide adequate individual distances between the horses.

**Methods:**

To do so, we assessed behavioral and physiological parameters (via video behavioral observations and salivary cortisol measurements) of up to 28 horses in a loose housing system. Over 2 observation days per treatment, four AFRs were investigated in a balanced sequence: 1:1.2, 1:2, 1:3, and C (single feeding in familiar surroundings as a control).

**Results:**

We found that the horses expressed less aggressive behavior, especially those behaviors with a high risk of injury such as biting and kicking, when there were three times as many openings as there were horses at time-controlled hay racks, as compared with only 20% more openings or twice as many openings as there were horses [lineal mixed model: *F*_(3, 4)_ = 7.411; adjusted *R*^2^ = 0.733; *p*_(AFR_1:2)_ = 0.06, *p*_(AFR_1:3)_ = 0.02, *p*_(AFR_C)_ = 0.01]. The salivary cortisol levels during feeding decreased more strongly with more generous AFRs [*p*_(AFR_metric)_ = 0.02]. The factors hierarchy and individual showed no influence. In contrast, the day of the experiment and the associated weather conditions, despite randomized selection, influenced both the behavioral and the physiological parameters.

**Discussion:**

The results of this preliminary study indicate that the investigated time-controlled hay racks must provide at least three times as many feeding places as there are horses to ensure that neighboring horses can keep their individual distance and stress-free feeding is possible. Further studies on more farms and different types as well as arrangement of hay racks are proposed.

## 1. Introduction

Automated feeding systems offer advantages for animal welfare and economic efficiency. Thus, they are increasingly utilized in housing systems for horses as well as other agricultural farm animals. For some farm animal species, such as pigs and rabbits, regulations for rationed feeding systems require that each animal has a feeding place available. This feeding place must be designed in a way that allows all animals to eat simultaneously. Thus, for pigs, for example, the German animal welfare legislation stipulates a minimum feeding place width per weight class and age (1, 2). The Swiss Animal Welfare Ordinance does not contain any regulations on the number or design of feeding places, but every animal keeper must ensure that each animal receives sufficient feed (3). For horses, the German guidelines for the assessment of horse husbandry systems under animal welfare aspects stipulate the following requirements (4): “Each horse, also in group living, must always have a feeding place available. If this is not the case (e.g., computer-controlled feeding), appropriate measures must be taken to ensure that all horses can eat at least roughage simultaneously.” For horses, feeding systems with and without partitions between the horses are available. High partitions allow undercutting the individual distance, and thus a feeding place width of only about 80 cm per horse is possible. Specific information on the minimal feeding place width in group housing systems is only provided for feeding stands with partitions (4). However, the prescribed feeding place width of 80 cm per horse cannot be used as minimum feeding place width at time-controlled hay rack because here, protection from the neighboring horse is not given. Without protective partition, the individual distance of horses during feeding can be up to several meters, depending on compatibility and food supply (5). The adequate animal-to-feeding-place ratio (AFR) for feed stands without partitions (time-controlled hay racks) was never investigated.

Animals are sentient beings with the ability to experience various affective states. The assessment and evaluation of the affective states of animals is of great importance for caretakers to meet the animals' requirements and needs regarding housing and handling (6–9). An apparent, observable expression of an animal's internal emotional state is, among other things, the animal's behavior (e.g., changes in body posture and facial expression) (10, 11). Horses, for example, rarely show aggressive behaviors in natural settings. However, their aggressiveness increases when they experience disturbances or resource restrictions (12). Thus, aggressive encounters between conspecifics can be an indicator of inappropriate group composition or resource scarcity (12). Apart from that, pain can cause horses to become lethargic or aggressive toward humans (13). Therefore, aggressive behavior serves as an indicator of poor welfare and thus of stress, pain, or suffering (14).

Not only their behavior but also their physiology allows conclusions about the welfare of animals (15). Stress responses, for example, can be determined by measuring salivary cortisol levels as a physiological marker of stress. In horses, salivary cortisol has been measured to investigate numerous stressful events such as transport, horseback riding, carriage drawing, lunging, behavior tests, weaning, and rehousing (16–23). Thus, salivary cortisol in combination with other indicators, such as behavior, provides valuable information on the stress level in horses.

Regarding the required AFR at time-controlled hay racks with feed-through panels for horses, so far only recommendations (24, 25) and manufacturers' suggestions are available. A scientific basis is missing. Therefore, the aim of this preliminary explorative field study was to investigate which AFR for a widely used type of time-controlled hay racks with a specific feeding rhythm would be necessary to allow horses a relaxed food intake, i.e., feeding without frequent aggressive encounters and preferably without a physiological stress response.

## 2. Materials and methods

### 2.1. Animals and test farm

**Figure 1:**
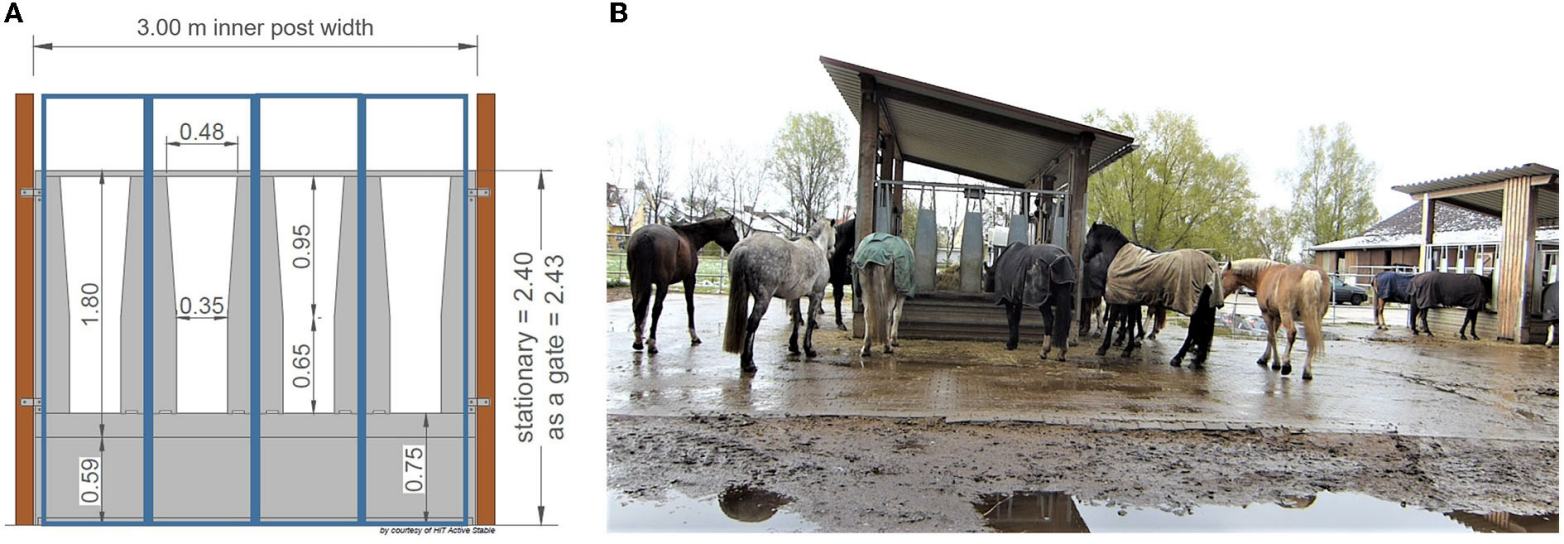
**(A)** Feed-through panel with four openings (^©^HIT Active Stable). The feeding place width per opening is 75 cm (300 cm/4; blue boxes). **(B)** Two time-controlled hay racks of different sizes on the test farm (^©^Baumgartner).

**Figure 2:**
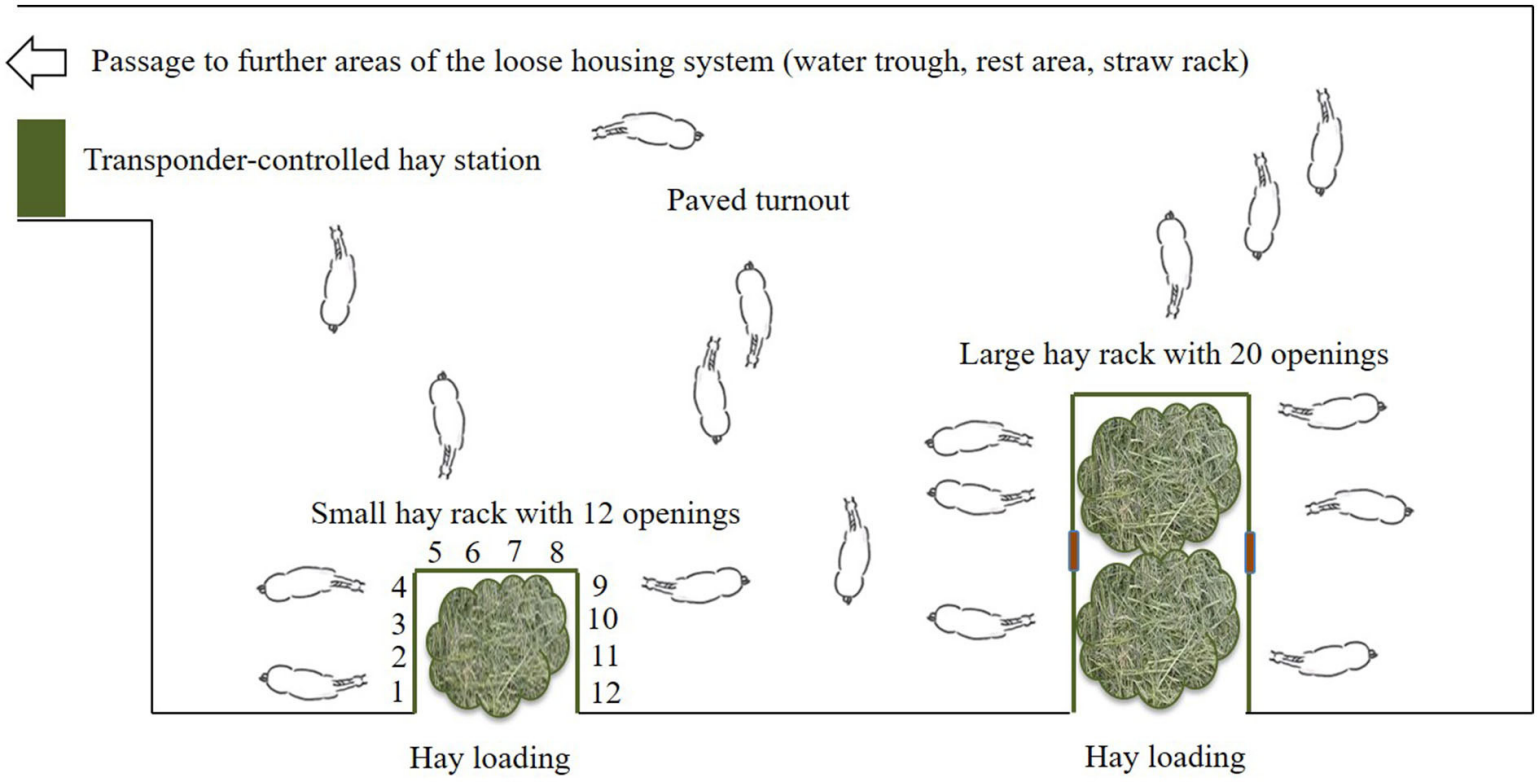
Sketch of the experimental area in the loose housing system (open horse stable with permanent access to a turnout area) with a total of 32 feed-throughs (openings) at two time-controlled hay racks (^©^Baumgartner).

The test site was a loose housing system with 28 sport and leisure horses of various breeds and ages (mean ± SD: 13.9 ± 4.8 years old; range: 6–23 years) and of both sexes (nine mares, 19 geldings). Routine care as well as daily equestrian use was dependent on each owner of the horses. None of the horses was a school horse. Two time-controlled hay racks with feed-through panels (32 openings, 75 cm feeding place width per opening) of the company HIT Active Stable provided automated hay supply (Figures 1, 2). In the following, we use the term opening or feed-through as a synonym for feeding place.

Except for the nighttime pause (midnight to 6:59 a.m.), the horses had access to the hay in a 2-h rhythm (access times: 7, 9, and 11 a.m., 1, 3, 5, 7, 9, and 11 p.m.), with an access duration of 28 min and blocked access for 92 min. Thus, the horses had access to hay for 4.2 h/day. Depending on the body condition score individual horses additionally received hay and concentrate feed from a transponder-controlled feed station. All horses were provided straw *ad libitum* in a non-controlled rack.

### 2.2. Behavior observations

The level of stress in the horses was assessed *via* behavioral and physiological parameters. The former included aggressive behaviors with low and high risk of injury (Table 1). These behaviors were counted as individual actions. In case of interactions that included displacement from the feed-through, only the result (i.e., displacement) along with the associated level of aggression was recorded. For the behavior observations, individual animals were not distinguished.

**Table 1:** Categories of the behavioral parameter “aggressive behaviors” [modified after (25, 26)].

**Aggressive behavior**	**Risk of injury**	
	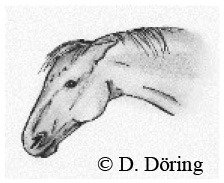	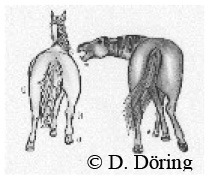
	**Low**	**High**
Facial threat expression (definition: ears pinned backward, ear openings facing backward, lips retracted, and nostrils narrowed)	X	
Head-swing threat (definition: facial threat expression and head swinging in the direction of the recipient without moving the body)	X	
Bite threat (definition: facial threat expression, mouth does not touch body of recipient)	X	
Kick threat with hindleg (definition: facial threat expression, leg not extended while kicking)	X	
Kicking with hindleg (definition: facial threat expression, leg extended while kicking)		X
Biting (definition: strong facial threat expression, mouth touches body of recipient)		X
Attacking (definition: strong facial threat expression, aggressor moves toward another animal)		X
Displacement from feed-through with low risk of injury (definition: facial threat expression and a threat gesture with low level of aggression that is associated with a low risk of injury for the recipient)	X	
Displacement from feed-through with high risk of injury (definition: facial threat expression and a threat gesture with high level of aggression that is associated with a high risk of injury for the recipient)		X

The aggressive behavior of the horses at the time-controlled hay racks was analyzed in two replicates per treatment (see Section 2.4) in a balanced sequence during the same feeding phase or daytime per observation day. The observation period began when the racks were opened for food intake and ended when they were closed (28 min of access authorization = 28 min of observation). The analysis of the behavior observations was based on video recordings with continuous behavior sampling according to Martin and Bateson (27).

### 2.3. Salivary cortisol measurements

As physiological stress parameter, we determined salivary cortisol concentrations before and during feeding in 10 quasi-randomized horses (three horses of low, four horses of intermediate, three horses of high social dominance hierarchy as assessed by the farm manager). These 10 horses were on average 14.5 ± 4.9 years old and included three mares and seven geldings. Salivary cortisol samples (*n* = 236) were collected at predefined times, twice before and twice during feeding to measure two base values and two stress values, respectively, of cortisol concentration. For sampling, the horse was put on a halter and held calmly for ~1 min, so it could, if necessary, finish chewing on hay that it had already taken up. Saliva sampling and processing was done as described by Schmidt et al. (17) and Ishizaka et al. (28), p. 82: “The Salivette was inserted at the angle of the lips into the mouth of the horse and placed gently onto the tongue for 1 min until it was well-soaked. After centrifugation for 10 min at 1,000 *g*, 1 mL of saliva was aspirated, transferred into polypropylene tubes (Sarstedt), and frozen at −20°C until analysis. […] Cortisol concentration was determined with a commercial enzyme immunoassay validated for equine saliva.” By always sampling at the same daytime (i.e., during the same feeding phase), the circadian rhythm could be considered. In addition, a diurnal cortisol profile was determined for each horse.

**Figure 3:**
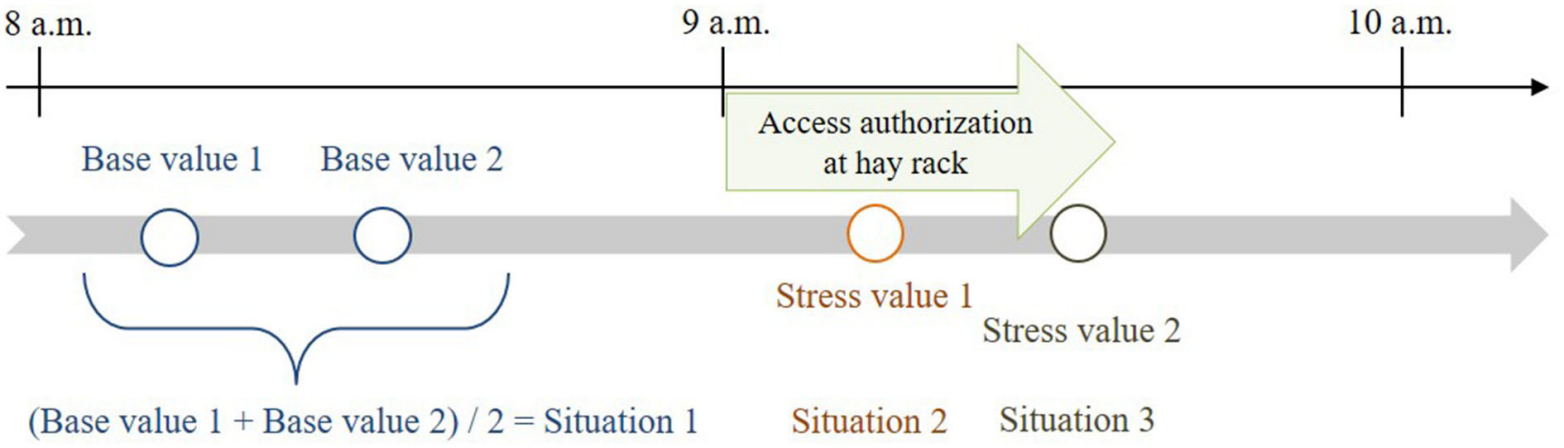
Times of salivary cortisol sampling in relation to feeding times (feeding phase: access authorization at the time-controlled hay racks).

The two base values were determined for each horse ~50 and 30 min before feeding. The mean value of these two base-value samples served as reference value for the calculations and was compared with stress values 1 and 2. Stress value 1 was determined ~15 min after opening and stress value 2 ~20 min later during closing of the hay rack feed-throughs (Figure 3), considering a delay of ~10 min in the diffusion of cortisol after a stressful event (22, 42). In total, 350 saliva samples (10 horses × 4 values × 8 observation days; plus 30 diurnal cortisol profile values) were taken and processed for the analysis.

### 2.4. Test procedure

The study included four treatments that differed regarding the provided AFR, namely, 1:1.2 with 20% more feed-throughs than horses (a recommendation from industry), 1:2 with twice as many feed-throughs as horses, 1:3 with three times as many feed-throughs as horses, and a control (=C) with single feeding in the grooming area with familiar conspecifics nearby. The different AFRs (for the whole group of 28 horses) were created by the quasi-randomized removal of 2 (AFR of 1:1.2), 12 (AFR of 1:2), or 18 horses (AFR of 1:3) from the loose housing system 30 min before access authorization to the hay racks. The horses were used to daily fluctuation of the group composition, as horses were taken out of the group for riding by their owners every day and at different times, even during feeding. The removed horses did not belong to the 10 horses sampled for salivary cortisol measurements. Randomized selection of the treatments resulted in the following sequence of 8 observation days, which extended over a period of 3 weeks: day 1 = C, day 2 = 1:3, day 3 = 1:1.2, day 4 = C, day 5 = 1:1.2, day 6 = 1:3, day 7 = 1:2, and day 8 = 1:2. A preliminary test was conducted to habituate the horses and to try out the procedure.

### 2.5. Statistical analysis

The descriptive and graphical evaluation of the data was done in (44). For the statistical analysis, linear mixed-effects models (LMM) and generalized linear mixed models (GLMM) were employed in R (29). The models analyzed the influence of treatment (i.e., AFR) on the dependent variable “number of aggressive behaviors.” Observation day was included as a random effect in the model for the behaviors with low risk of injury but not in the model for the behaviors with high risk of injury because these occurred at low frequency (multiple zero values).

For the physiological parameter, the models tested whether the mean value of the two salivary cortisol base values was similar for each treatment. Thereby, it was checked whether the experimental conditions were the same for all treatments. Furthermore, the influence of the interaction between treatment and situation was analyzed. Situation 1 was defined as the mean salivary cortisol base value (see Figure 3) before feeding and was compared with situation 2 (stress value 1) and situation 3 (stress value 2) during the feeding phase. For this analysis, the categorical and the respective metric AFRs were considered (e.g., 1:1.2 ≜ 0.83; 1:3 ≜ 0.33) to determine a general and a specific influence, respectively, of the different AFRs. Additional fixed effects in the model were the dominance hierarchy of the horses and the observation day. As a random effect, repeated measurements of the horses per observation day were considered in the model as follows: GLMM (cortisol ~ situation^*^AFR_metric + dominance hierarchy + observation day + [AFR_metric|horseID], data = xy). The level of significance was set at α = 0.05.

### 2.6. Ethics statement

Horse farm managers voluntarily participated in the study. The study complies with the GDPR regulations, because the data is anonymized. This study was non-invasive. The study complies with the Guidelines for Ethical Treatment of Animals in Applied Animal Behavior and Welfare Research (43). Ethical review and approval was not required for the animal study because this study was non-invasive.

## 3. Results

### 3.1. Behavior observations

**Figure 4:**
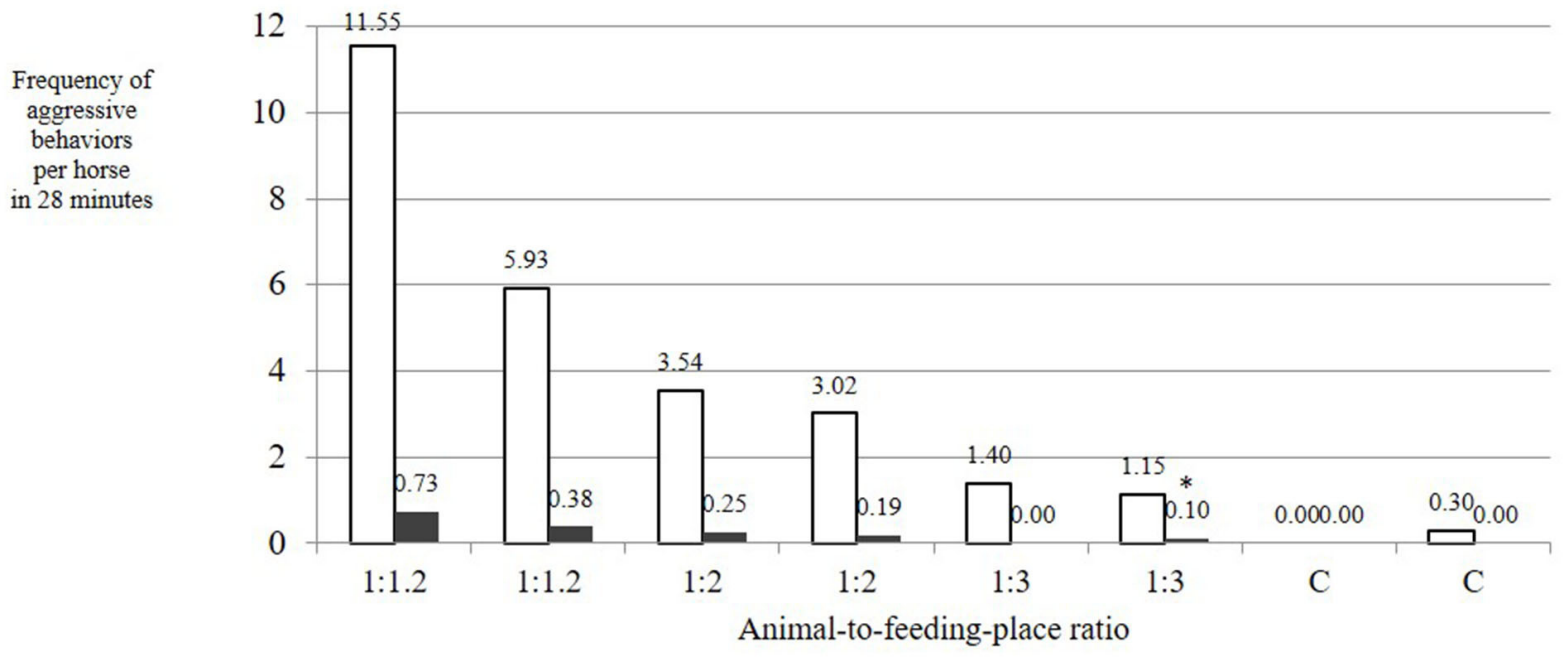
Frequency of aggressive behaviors per horse during a 28-min feeding phase at time-controlled hay racks with a varying animal-to-feeding-place ratio (1:1.2 = 20% more feed-through openings than horses; 1:2 = twice as many openings as horses; 1:3 = three times as many openings as horses; C = single feeding as a control; white bars = aggressive behaviors with low risk of injury; dark bars = aggressive behaviors with high risk of injury). Influence of AFR on the number of aggressive behaviors with low risk of injury: LMM, *F*_(3, 4)_ = 7.268; adjusted *R*^2^ = 0.729; *p* = 0.04. Influence of AFR on the occurrence of aggressive behaviors with high risk of injury: LMM, *F*_(3, 4)_ = 7.411; adjusted *R*^2^ = 0.733; *p* = 0.04, *p*_(AFR_1:3)_ = 0.02, *p*_(AFR_C)_ = 0.01.

The number of aggressive behaviors with low risk of injury per horse and feeding phase was dependent on the AFR. These behaviors showed a linear decrease with increasing availability of feeding places (mean ± SD over both observation days per treatment: 1:1.2: 8.74 ± 2.81; 1:2: 3.28 ± 0.26; 1:3: 1.27 ± 0.13; C: 0.15 ± 0.15 per horse in 28 min; Figure 4). This influence of treatment on the number of aggressive behaviors with low risk of injury was statistically significant [LMM: *F*_(3, 4)_ = 7.268; adjusted *R*^2^ = 0.729; *p* = 0.04]. The difference was particularly evident for treatments AFR 1:3 and C if compared with the reference category 1:1.2 [*p*_(AFR_1:2)_ = 0.05, *p*_(AFR_1:3)_ = 0.02, *p*_(AFR_C)_ = 0.01]. If the model included observation day as a random effect, the differences of AFR 1:2 and AFR 1:3 from AFR 1:1.2 were tendential [LMM fit by restricted maximum likelihood; df = 4; *p*_(AFR_1:2)_ = 0.13, *p*_(AFR_1:3)_ = 0.06, *p*_(AFR_C)_ = 0.04], associated with a large difference between the frequencies of aggressive behaviors with low risk of injury on the 2 observation days of treatment AFR 1:1.2 (Figure 4).

The aggressive behaviors with high risk of injury such as biting and attacking exclusively occurred in the treatments with low AFRs (1:1.2 and 1:2), not in AFR 1:3 or in the control. The influence of treatment (i.e., AFR) on the occurrence of aggressive behaviors with high risk of injury was statistically significant [LMM: *F*_(3, 4)_ = 7.411; adjusted *R*^2^ = 0.733; *p* = 0.04; *p*_(AFR_1:2)_ = 0.06, *p*_(AFR_1:3)_ = 0.02, *p*_(AFR_C)_ = 0.01]. Overall, aggressive behaviors with high risk of injury occurred at a low level (mean ± SD: 0.02 ± 0.24; median: 0.15; range: 0–0.73 occurrences per horse in 28 min).

### 3.2. Salivary cortisol measurements

**Figure 5:**
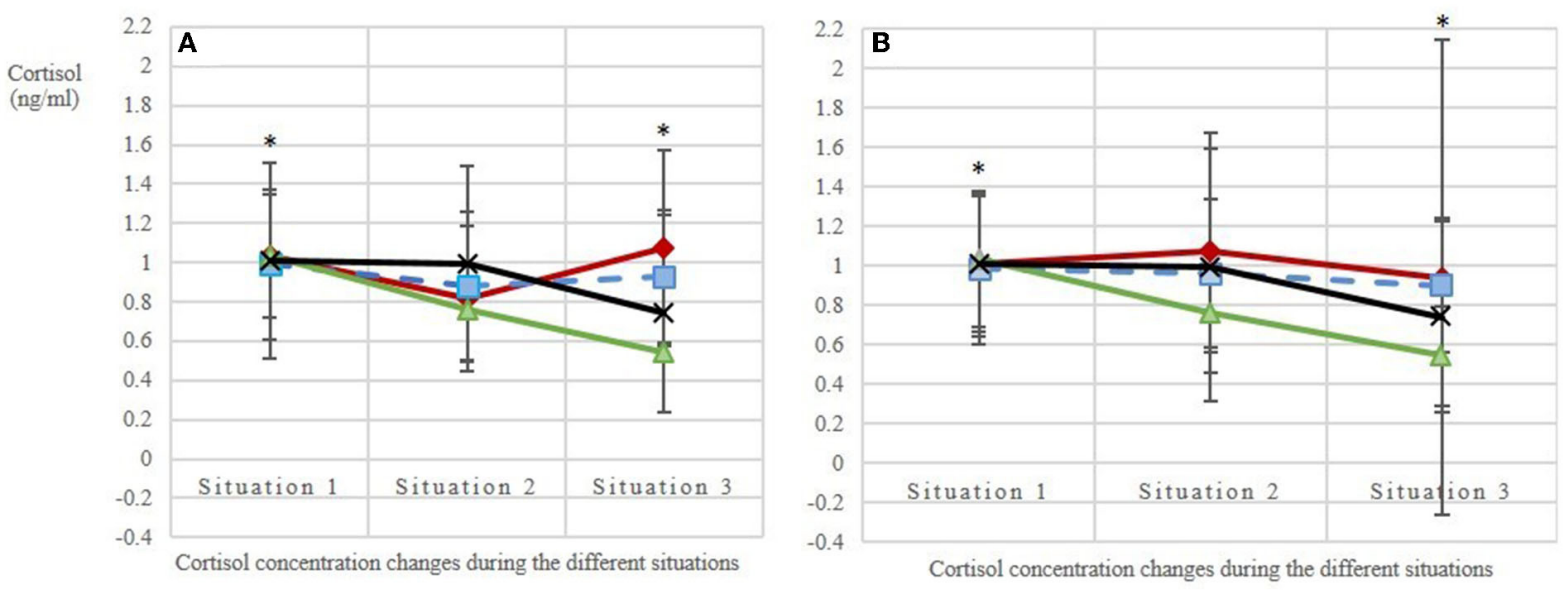
Salivary cortisol levels **(A)** in the first replicate (observation days balanced, the 1st day of each treatment is given: days 3, 7, 2, 1; *N* = 10 horses; *n* = 116 analyzed saliva samples) and **(B)** in the second replicate (observation days balanced, the 2nd day of each treatment is given: days 5, 8, 6, 4; *N* = 10 horses; *n* = 120 analyzed saliva samples). Situation 1: base value before feeding; situations 2 and 3: stress values 1 and 2, respectively, during feeding with an interval of ~15 min. The treatments included three animal-to-feeding-place ratios (red line with ♢ = 1:1.2; blue dashed line with | = 1:2; green line with ▴ = 1:3) and a control (black line with X = C, single feeding in the grooming area). Stars mark the statistically significant decrease of salivary cortisol concentrations (Δ) between situations 1 and 3 in treatments AFR 1:3 and C as compared with the reference category 1:1.2 [LMM: $p_{\left(\text{situation}3^{*}AFR\text{\_}1:3\right)}$ = 0.02; $p_{\left(\text{situation}3^{*}AFR\text{\_}C\right)}$ = 0.03].

The base values of salivary cortisol concentration before the beginning of each of the four treatments (situation 1 per treatment) did not differ [mean base value ± SD per AFR: 1:1.2 = 1.03 ± 0.52; 1:2 = 0.85 ± 0.30; 1:3 = 0.99 ± 0.54; C = 1.03 ± 0.57 ng/ml cortisol; *n* = 80 values; *N* = 10 horses; LMM: adjusted *R*^2^ = 0.01; *F*_(3, 76)_ = 1.44; *p* = 0.24; Figure 5], confirming that the experimental conditions were the same for all treatments. Over the course of the measurements (i.e., from the base value to stress value 2), the salivary cortisol concentrations (Δ) decreased more strongly with increasing AFRs (*p*_(AFR_metric)_ = 0.02). The two additional effects dominance hierarchy and individual did not influence these cortisol concentration changes observed between the different situations, whereas the observation day had an effect on the cortisol concentration (*p*_(observationday)_ = 0.01). By using the categorical AFRs in the model, we could show that the difference between situations 1 and 3, i.e., the mean base value and stress value 2, was statistically significant in treatments AFR 1:3 and C as compared with the reference category 1:1.2 ($p_{\left(\text{situation}3^{*}AFR\text{\_}1:3\right)}$ = 0.02; $p_{\left(\text{situation}3^{*}AFR\text{\_}C\right)}$ = 0.03).

The same holds true for the additionally determined diurnal cortisol profiles of the horses: Despite the tendentially decreasing cortisol concentrations from morning (mean ± SD: 0.85 ± 0.27 ng/ml cortisol) to midday (0.67 ± 0.22 ng/ml cortisol) and evening (0.68 ± 0.24 ng/ml cortisol), the values showed no statistically significant differences [*n* = 28 analyzed cortisol values; *N* = 10 horses; adjusted *R*^2^ = 0.13; *F*_(4, 23)_ = 1.99; *p* = 0.13]. The dominance hierarchy, included as a potential additional effect in the LMM, did not influence the diurnal cortisol profiles.

## 4. Discussion

Automated feeding systems support farm managers in feeding the horses as species-appropriately as possible (short feeding interruptions, long periods of roughage feeding) while at the same time limiting the access to food to allow needs-based feeding and, thus, prevent obesity of the horses due to excessive energy intake. Therefore, automated feeding systems are increasingly used on horse farms. However, besides offering advantages for animal welfare, computer-controlled feeding can cause problems such as increased aggression levels, blocking or urinating in transponder-controlled feed stations, or constrained back and neck postures (24, 30–34).

Briefer et al. (24) compared different feeding frequencies at a time-controlled hay rack with an animal-to-feeding-ratio (AFR) of 1:2. They found higher levels of aggression in horses when the hay rack was opened six times a day, than when it was opened only three times a day. An undisturbed roughage intake at the hay rack with an AFR of 1:2 was never observed. Briefer et al. (24) therefore suggested to carefully evaluate whether the AFR is sufficient and the group composition is homogenous. To date, no literature determining the ideal AFR ratio is available. The present study aims to close this research gap by investigating the adequate AFR for time-controlled hay racks. An explorative field experiment in a loose housing system with time-controlled feed-through hay racks served to determine the minimum AFR required to allow a stress-free roughage intake of horses feeding at hay racks.

Aggressive behaviors in horses are rarely observed in a natural setting, because horses prefer to socially communicate with subtle non-aggressive behaviors such as avoidance or retreat (12–14, 25). In equine husbandry systems however, aggressive behaviors disturb the feeding behavior of horses and pose a greater risk of injury. Furthermore, aggressive behaviors mirror conflict, pain or discomfort in the addressor (14, 25, 35–37). Hence, the frequency of aggressive behaviors, regardless of the level of the resulting injury, reflects the welfare of all horses involved. According to the Swiss Animal Welfare Act wellbeing of animals only exists if pain, suffering, harm and anxiety are avoided (3). A high frequency of aggressive behaviors presents such sign of stress, pain, or suffering (14, 38). The current study reports aggressive behaviors associated with high risk of injuries, such as biting and kicking, almost exclusively in small AFR of 1:1.2 and 1:2. Our results suggest that horses show lower levels of aggression if the number of feeding places at time-controlled hay racks clearly exceed the number of horses present. Thus, the ethological parameters are indicative of a stressful and potentially harmful housing condition. Our findings demonstrate that increasing AFR would avoid such harm and suffering.

The physiological parameter salivary cortisol confirmed the behavioral parameter in that a more generous AFR led to a stronger decrease in salivary cortisol concentration during feeding. This was especially true for the AFR of 1:3 and the control (single feeding in the grooming area) in comparison with the AFR commonly provided in practice (i.e., 1:1.2). However, we also found a confounding effect of observation day, despite randomized selection of the observation day. This effect was obvious during one of the observations days of AFR 1:1.2, when a change in weather condition may have led to markedly increased restlessness associated with higher stress responses in the horses. Even though the herein assessed behavioral and physiological stress responses point in the same direction, the use of salivary cortisol concentration as a stress parameter in feeding experiments may be questionable. Compared with concentrations reported for other stressful events, such as transport, horseback riding, carriage drawing, lunging, behavior tests, weaning, and rehousing [ranging from about 0.5 to about 8 ng/ml cortisol; (16–23)], the concentrations measured in the present study are low (about 1 ng/ml). So far, only a few studies have also found an unchanged or decreasing salivary cortisol level (28, 39). An explanation for the low cortisol concentrations could be the activation of the parasympathetic nervous system during feeding, as demonstrated by heart rate variability measurements in horses during grazing on pasture (40). The stress-reducing effect of eating could mask the effects of the various AFRs. Another explanation for the low cortisol concentrations might be a habituation effect because the horses had been used to the feeding situation for a long time, although aggressions were high during the commonly used AFR. For example, Schmidt et al. (16) found that horses during repeated transport showed a decrease in salivary cortisol concentration because of stress reduction as a result of habituation. Finally, cortisol concentrations are much lower in saliva than in plasma; therefore, as done in our study, changes should be interpreted intra-individually (41).

To allow a general statement on the optimal AFR for horses, future studies should consider variations across horse farms with different housing and management conditions especially different types of hay racks and different group compositions regarding sex, age, and roughage requirement of the horses. Furthermore, despite quasi-randomized selection and repetition, the variation in group composition in studies such as ours is a critical aspect because group dynamics can influence social interactions or aggressive encounters. As an alternative to our study setup, the number of feed-throughs could be increased by installing additional time-controlled hay racks on the farm. To modify the AFRs for the experimental treatments, the non-required feed-throughs on one side of a hay rack could remain permanently closed instead of having to reduce the number of horses in the group. To maintain the individual distances in such a setup, the non-accessible feed-throughs must not be between the accessible ones; otherwise, the individual distances would differ between treatments, and thus the experimental conditions would not be comparable. In the presented explorative field study, the provided AFR of 1:1.2 was considered the least favorable variant, and the purchasing of additional time-controlled hay racks was impossible owing to building regulations and financial reasons.

## 5. Conclusion

In conclusion, our results question the frequently found conditions of only 20% more feeding places (feed-throughs and openings) than horses in loose housing systems with the investigated type of time-controlled hay racks. Our explorative study showed that for this type of rack and this feeding rhythm aggressive behaviors associated with a high risk of injury, such as biting, kicking and attacking, exclusively occurred in the treatments with small AFRs (1:1.2 and 1:2), and were not found in the more generous AFR of 1:3 or in the control. The salivary cortisol measurements confirm these ethological findings since only treatments AFR 1:3 and C reflected low levels of cortisol during feeding, whereas cortisol levels in AFR 1:1.2 and 1:2 were elevated. At the investigated time-controlled hay racks, at least three times as many feeding places as horses seem to be necessary to ensure the individual distance to the closest neighbor so that, according to the assessed behavioral and physiological parameters, the horses are mostly relaxed during hay intake. Further studies are needed to validate the results.

## Data availability statement

The raw data supporting the conclusions of this article will be made available by the authors, without undue reservation.

## Ethics statement

Ethical review and approval was not required for the animal study because this study was non-invasive. The study complies with the Guidelines for Ethical Treatment of Animals in Applied Animal Behavior and Welfare Research (ISAE Ethics Committee, 2017). Written informed consent was obtained from the owners for the participation of their animals in this study.

## Author contributions

MB and MZ-F conceptualized the study and acquired funding. MB carried out the practical data collection on farms, performed the formal and statistical analyses, drafted the manuscript, and edited the paper. MB, ME, and MZ-F revised the paper. All authors have read and agreed to the published version of the manuscript.
